# Early life history of the Korean seahorse, *Hippocampus haema*: Evidence for direct settlement and ontogenetic shifts in snout morphology

**DOI:** 10.1111/jfb.70593

**Published:** 2026-07-24

**Authors:** Jaehwan Juan Lee, Jin‐Koo Kim

**Affiliations:** ^1^ Department of Marine Biology Pukyong National University Busan Korea; ^2^ Present address: School of Biological Sciences, University of Canterbury Christchurch New Zealand

**Keywords:** early history, early settlement, morphological development, reproductive strategy, Syngnathidae

## Abstract

Early life‐history traits of seahorses are closely linked to juvenile survival, dispersal potential and the onset of substrate attachment; however, detailed stage‐resolved descriptions remain limited for many *Hippocampus* species. This study examined reproductive behaviour and early ontogenetic development of the Korean seahorse, *Hippocampus haema*, during a 40‐day laboratory rearing period. Newborns were released in a relatively advanced skeletal condition, particularly in the caudal skeleton. Skeletal staining showed that the caudal bony plates were already ossified and connected at birth, whereas the trunk plates were still only partially connected. Consistent with this condition, newborns bent the prehensile tail and attached to nearby structures immediately after release, indicating functional competence for substrate attachment at birth and suggesting a very short or indistinct planktonic phase under the observed conditions. Coronet morphology changed markedly during juvenile development, with the anterior coronet spine progressively fusing with the coronet. Morphometric model comparison did not strongly support a continuous power function allometry across measured traits. However, segmented regression identified a significant breakpoint in snout depth at 21.3 mm standard length, after which snout depth increased approximately 2.6‐fold more steeply. These findings indicate that *H. haema* is characterised by advanced caudal skeletal development at birth, immediate tail attachment, dynamic coronet development and a discrete ontogenetic shift in snout depth growth. This stage‐resolved description provides baseline information for identifying early developmental stages and interpreting early life history variation in this species.

## 1 INTRODUCTION

The family Syngnathidae, which includes seahorses, pipefishes and seadragons, is a very diverse family of fishes, comprising approximately 309 recognised species (Froese & Pauly, 2026; Hamilton et al., 2017). Members of this family share distinctive traits, such as an armoured body with bony plates, an elongated snout, the absence of pelvic fins and male pregnancy (Kuiter, 2009; Vincent, 1996). Within syngnathids, seahorses refer to fishes of the genus *Hippocampus*, with 48 species currently recognised worldwide (Froese & Pauly, 2026; Koning & Hoeksema, 2021). Owing to their limited swimming ability, small home ranges and low fecundity, seahorses are particularly susceptible to human‐driven disturbances, including habitat degradation, bycatch and overfishing (Foster & Vincent, 2004). Accordingly, all seahorse species are listed in appendix II of the Convention on International Trade in Endangered Species of Wild Fauna and Flora (CITES), under which their international trade is regulated (CITES Secretariat, 2026). As of December 2025, global assessments indicate that at least 16 seahorse species are threatened (Critically Endangered, Endangered or Vulnerable) on the IUCN Red List, while many others remain Data‐Deficient (IUCN, 2025).

Early life history in fishes is a critical stage that determines survival and dispersal, and variation in early‐stage phenotypes and behaviours can strongly influence recruitment success (Chambers & Trippel, 1997; Houde, 1987). Because adult seahorses have limited dispersal ability, early life traits such as the duration of the planktonic larval phase, the timing of settlement, size at birth and brood size can be more important determinants of population connectivity and patterns of habitat use in seahorses than in many other fish species (Curtis et al., 2017; Curtis & Vincent, 2006; Foster & Vincent, 2004; Harasti et al., 2012; Morgan & Vincent, 2013). These traits are not consistently correlated even among species within this genus. For example, although *Hippocampus barbouri* and *H. hippocampus* have similar maximum adult lengths (15 cm), they differ in both newborn size and brood size: *H. hippocampus* produces larger newborns (9.3 mm) and a larger brood size (*n* = 865) than *H. barbouri* (5.0 mm; *n* = 250) (Lourie et al., 2004; Miller & Lea, 1972). This pattern suggests that early life traits and reproductive strategies vary among species and may reflect differences in habitat conditions and life history strategies (Foster & Vincent, 2004). Therefore, investigating phenotypic and behavioural changes during early development at the species level can provide useful starting points for discussing ecological vulnerability and conservation needs (Hobbs et al., 2019; Vincent et al., 2011).

The Korean seahorse, *Hippocampus haema*, is distributed along the southern and eastern coasts of Korea and was described as a new species after taxonomic revision of the *H. coronatus* complex. Morphological and molecular evidence supported its distinct evolutionary position from closely related species (Han et al., 2017). Since then, studies on *H. haema*, such as ecology, feeding, parasites and reproduction have increased, and potential population declines linked to its restricted habitat and rapid coastal environmental change have been discussed (Choi et al., 2012; Huh et al., 2014; Kim et al., 2022; Park & Kwak, 2015). Taken together, these studies indicate that *H. haema* relies largely on nearshore structured habitats, such as seagrass beds (Cho et al., 2022). Because these habitats are easily fragmented, successful settlement during early life is expected to be critical for population persistence (Foster & Vincent, 2004; Vincent et al., 2011). However, most previous studies have relied on field‐collected samples representing different developmental stages (Choi et al., 2012; Kim et al., 2018) or have provided only basic information on growth and survival under captive conditions (Choi et al., 2006). Moreover, seahorse juveniles rely heavily on live zooplankton prey during early development, which makes captive rearing particularly challenging (Payne & Rippingale, 2000). This limitation hampers detailed observation of early life behaviour and putative changes in survival strategies, and time‐resolved accounts of these stages remain limited.

The coronet is one of the most variable cranial structures among seahorse species and is often used as an important diagnostic character for species identification (Han et al., 2017; Lourie et al., 1999). However, coronet morphology may not be fully developed during early ontogeny and can change substantially during juvenile growth, which may limit its reliability for identifying early life stages (Choo & Liew, 2006; Kanou & Kohno, 2001; Novelli et al., 2017). This is particularly relevant for the *H. coronatus* complex, to which *H. haema* belongs, because closely related species such as *H. coronatus* and *H. sindonis* can be difficult to distinguish using a limited set of external or meristic characters (Han et al., 2017). Therefore, documenting the developmental sequence of the coronet, cephalic spines and bony plates in *H. haema* can provide essential baseline information for interpreting early‐stage field records and for future comparative studies within the *H. coronatus* complex. Such stage‐based morphological descriptions are also important for field monitoring, distributional assessment and recruitment studies, especially for species of conservation concern, because accurate identification of early stages is necessary for tracking spawning ground use and variability in early recruitment (Hobbs et al., 2019; Vincent et al., 2011).

Beyond their value for identification, morphological changes during early life may also reflect functional transitions rather than merely the outcome of growth. For example, in seahorses, suction feeding makes snout morphology, including snout length and snout depth, particularly relevant to feeding performance (Leysen, Dumont, et al., 2011; Roos et al., 2011). These traits may also influence microhabitat use within structured environments and the size range of prey that can be consumed (Kendrick & Hyndes, 2005). Accordingly, morphometric ratios and size‐related relationships are useful for quantitatively describing morphological changes during early development and for identifying shifts in the growth priorities of functionally important traits (Fuiman, 1983; Osse & Van den Boogaart, 2004). In addition, statistical approaches that detect slope changes (breakpoints) along growth trajectories can help identify potential ‘transition points’ in development (Davies, 1987; Muggeo, 2008). Such analyses can provide a basis for linking morphological change to functional interpretation.

This study aimed to clarify the early life history and ontogenetic morphological changes in *Hippocampus haema* under laboratory conditions. Specifically, we sought to characterise early morphological and behavioural traits associated with development and settlement, including bony plate formation, pigmentation patterns, tail bending and attachment behaviour. We also examined ontogenetic changes in morphometric relationships and examined whether breakpoints occur in these growth patterns, thereby exploring potential transitions relevant to early life ecological functions. Third, we provide stage‐specific morphological information for larvae and early juveniles, including the temporal sequence of coronet and spine development, to establish baseline data for interpreting early developmental stages of *H. haema* and for future comparative studies with closely related species. Overall, these results provide a stage‐resolved baseline for early‐stage identification of *H. haema* and for interpreting species‐specific early life morphology and behaviour in an ecological and conservation context.

## 2 MATERIALS AND METHODS

### 2.1 Broodstock sampling and breeding

Three pairs of adult *H. haema* were collected using a hand net from Port Namcheon (Suyeong gu, Busan, Korea) in August 2020. Sampling was restricted to small eelgrass patches within the inner harbour, where currents were weak and over muddy substrate at a depth of 1–2 m. The individuals were transported to the Pukyong National University Ichthyology laboratory and held in a glass tank (60 × 30 × 30 cm). The fish tank was continuously supplied with physically and biologically filtered water in a recirculating system. Plastic artificial plants and plastic nets were placed for seahorses to hold on to their tail. The temperature and salinity in the glass tank were maintained at 18 ± 1°C and 34 ± 2 psu, respectively. The fish tank was maintained under a 14 h light and 10 h dark photoperiod provided by 30 W incandescent light lamps. Adult seahorses were fed frozen mysid shrimp (Bio‐pure, Hikari Sales Inc., Hayward, CA, USA) and copepods (*Tigriopus* sp.) twice daily (at 9 AM and 7 PM) to satiation. The frozen mysid shrimp were fed to seahorses after thawing in water, and a wave maker pump (WP, Jebao, China) was used on the bottom to create water movement and keep the mysids in suspension. To supplement the nutritional limitations of the frozen mysids, adult seahorses were also fed copepods collected from the intertidal zone of Oryukdo (Namgu, Busan, Korea). Two hours after feeding, all excess food was removed to prevent decomposition.

### 2.2 Offspring breeding and observations

After mating, males showing a clearly distended brood pouch were considered pregnant. These males were immediately moved to a plastic container (24 × 11 × 15 cm), suspended near the upper part of the broodstock tank. Mesh screens (1 mm) were fitted to both sides of the container to allow offspring to flow into the broodstock tank. The tank conditions, including temperature and salinity, were the same as those for the adults. The offspring were fed *Artemia salina* nauplii (SepArt Technology, INVE Aquaculture, Dendermonde, Belgium) and rotifers twice a day (at 9 AM and 7 PM). For developmental observations, five individuals were sampled at 5 days before release, on the day of release (day 0) and at 4, 6, 8, 15, 20 and 40 days after release. Individuals were anaesthetised using MS‐222 and preserved in 99% ethanol. The preserved specimens were registered and deposited in the Ichthyology Laboratory collection of Pukyong National University (PKUI829‐PKUI836, Table S1). Juveniles were measured following Kuiter (2009) to the nearest 0.01 mm using a stereomicroscope (Olympus SZH10, Tokyo, Japan). Skeletal condition at birth was examined using specimens collected on the day of birth, which were stained with Alizarin Red S (Sigma‐Aldrich, St. Louis, MO, USA) to visualise ossified skeletal elements, following a modified procedure based on Taylor and Van Dyke (1985). The developmental stage and morphological terminology were defined according to Han et al. (2017) and Novelli et al. (2017), as illustrated in Figure 1.

**FIGURE 1:**
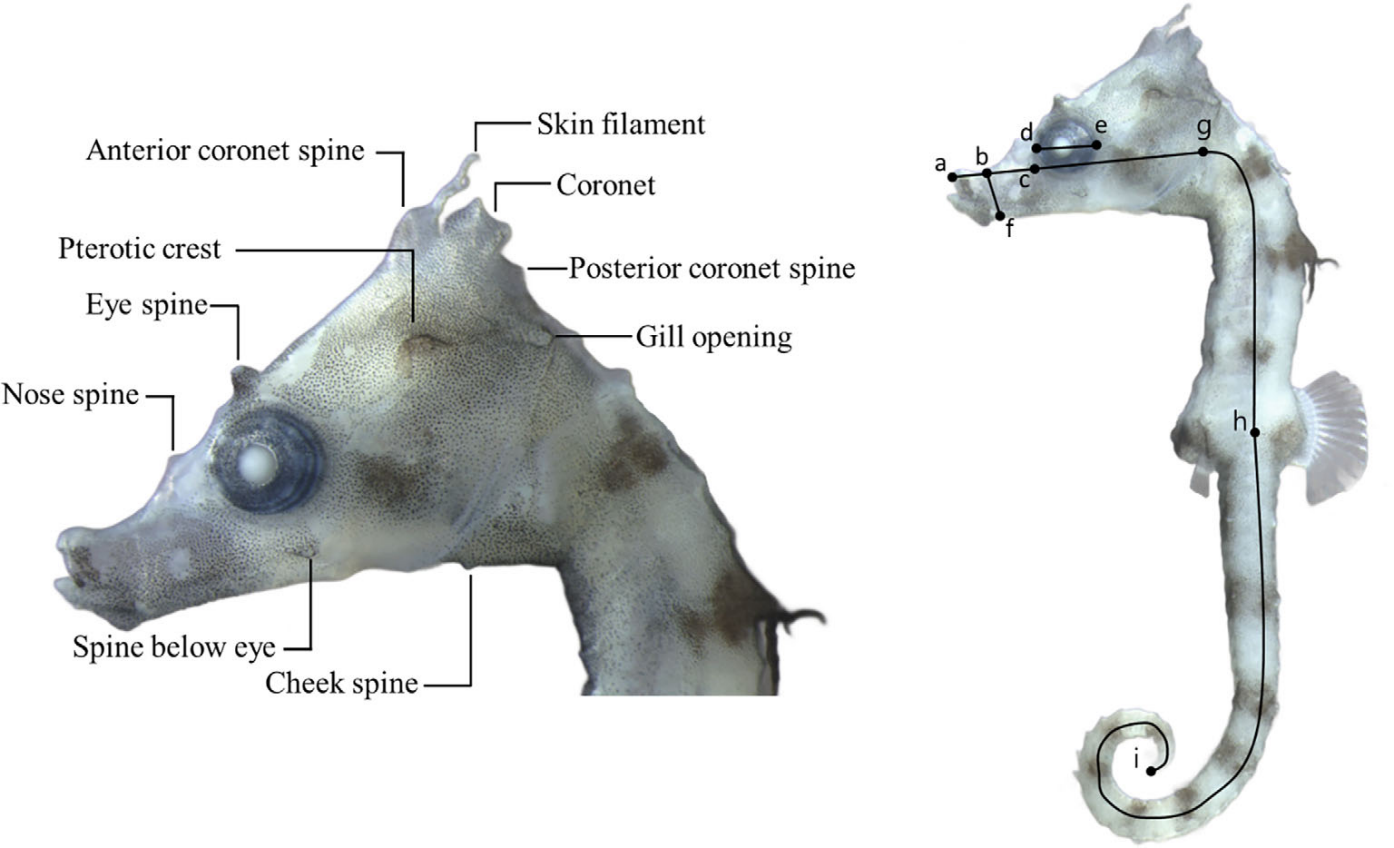
Meristic and morphometric characters. (a–c) Snout length, (b–f) snout depth, (d,e) eye diameter, (a–g) head length, (g,h) trunk length and (h,i) tail length. The photograph shows specimen DAB8 of *Hippocampus haema* (PKUI833‐1).

### 2.3 Data analysis

Day–length relationships were reported as the mean ± standard deviation (SD). The best‐fitting regression model was selected using curve estimation in SPSS version 25 (IBM, Armonk, NY, USA). Morphometric relationships between standard length (SL) and each measured character were evaluated using linear, power function and segmented regression models. Linear models were fitted as y=a+bx, whereas power function models were fitted as $y=ax^{b}$ (Fuiman, 1983). In these models, x is the SL, y is the measured character, *a* is the intercept in the linear model and the scaling coefficient in the power function model and *b* is the slope in the linear model and the scaling exponent in the power function model. Model support was compared using the corrected Akaike information criterion (AICc), with lower AICc values indicating stronger model support (Burnham & Anderson, 2002). Coefficients of determination (*R* ^2^) were used as supplementary measures of model fit. Power function relationships were not interpreted as evidence of allometric growth unless the power function model was better supported than the corresponding linear model. To identify specific points (breakpoints) in the relationship between the SL and each morphological trait, we applied Davies' test (Davies, 1987) using the R package segmented (R v4.3.0) (Muggeo, 2008), with 1000 resampling iterations and a significance threshold of *p* < 0.05. Breakpoints were interpreted only when Davies' test was significant. Five individuals sampled prior to parturition were excluded from the snout depth analysis. All figures were produced using ggplot2 in R.

## 3 RESULTS

### 3.1 Reproductive behaviour

Males showed darker colouration than females, including dark brown or orange tones. Their brood pouch became fully developed and opened in a behaviour known as ‘pumping’, which is associated with courtship and preparation for egg transfer (Woods, 2000). Courtship began when the male approached the female while pumping his brood pouch. He moved carefully towards her and maintained visual attention on her (Figure 2a). When the female did not respond, the male swam closer to attract her attention (Figure 2b). The pair then faced one another and rose vertically from the bottom while maintaining an upright posture (Figure 2c). The male grasped the female's tail with his own, and the pair swam together gently (Figure 2d). During mating, the male brought his open brood pouch towards the female's abdomen and aligned it with her ovipositor, allowing the eggs to be transferred into the pouch. Approximately three weeks after mating, brood size varied among males, ranging from 48 to 60 offspring per brood (54 ± 6.0 offspring; mean ± SD; *n* = 3 broods).

**FIGURE 2:**
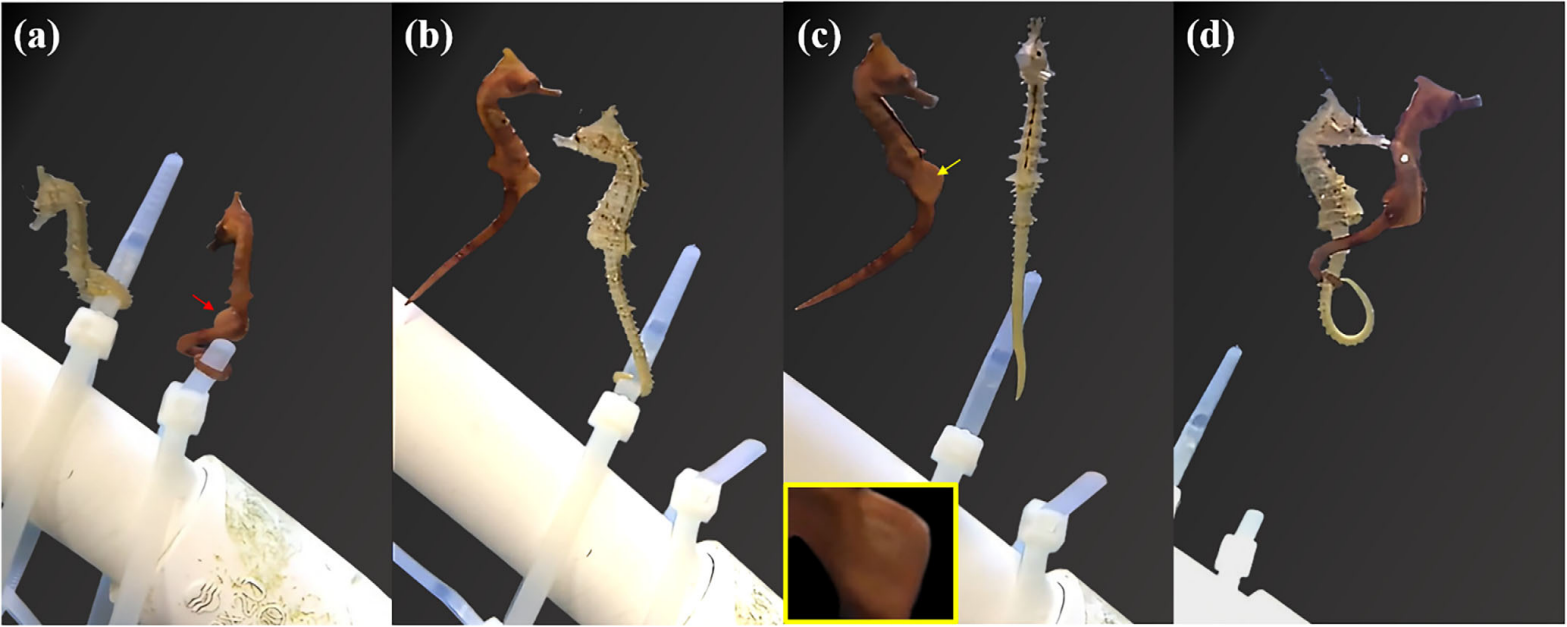
Reproductive behaviour exhibited by *Hippocampus haema*. (a) The male approached the female while pumping the brood pouch. (b) The male swam forward to the female. (c) The male and female rose vertically with the male brood pouch opened. (d) The male grasped the female's tail. The red and yellow arrows indicate the pumped brood pouch and the opened brood pouch, respectively.

### 3.2 Morphological development

Before the normal release of newborns, one male released several offspring from its brood pouch. Because the same male released normally developed newborns 5 days later, these prematurely released individuals were regarded as specimens corresponding to 5 days before release and were included in the analysis. Among normally released newborns from a single male, two pigmentation patterns were observed: a striped pigmentation pattern and extensive melanophore coverage over most of the body. Because these patterns occurred within the same brood at the same release event, they were not interpreted as representing different developmental stages. However, the extensively melanised type showed little stage‐related variation in pigmentation pattern and was therefore excluded from the stage description of melanophore development. Developmental stages were defined according to changes in the coronet, body plates, melanophores and head spines (Table 1).

**TABLE 1:** Summary of main morphological changes in *Hippocampus haema*.

Stage	Head spine	Coronet	Bony plates	Melanophore	Fin	Skin filament
Before release	Not developed	Round	‐	Small brown (nape, back, abdomen, tail)	Complete number of fin rays	‐
Day of birth	Eye, nose, and spine formed	Anterior coronet spine, coronet formed	Developed but not linked	Small black (extend head to tail)	Completely developed	‐
Day 4	Increase in size	Protruded upward	Touched each bony plate	More distinct	‐	‐
Day 8	Spine below the eyes formed	Posterior coronet spine formed	Body rings formed	Disappeared (nose and under the eyes)	‐	Formed on the body ring and anterior coronet spine
Day 15	‐	‐	Tail ring spines formed	Disappeared (body and tail except spine)	‐	Change in length
Day 20	Increase in size	Outgrew anterior coronet spine	Increase in size	‐	‐	Disappear completely
Day 40	Cheek spine buds	Connected with the anterior coronet spine	‐	‐	‐	‐

Offspring prematurely released from the brood pouch five days before the expected release measured 5.93–8.94 mm in SL (mean = 6.93 mm, *n* = 5) and retained a yellow yolk mass on the abdomen. The snout was relatively short, and the head was rounded. The ceratohyal bone protruded ventrally, and the neck was oriented nearly perpendicular to the body. At this stage, the full complement of pectoral, anal and dorsal fin rays had already developed (12, 4 and 14–15, respectively). Small brown pigmented spots were scattered over the nape, dorsum and abdomen. The tail was heavily pigmented, with striped pigmentation appearing every two to three tail rings (Figure 3a).

**FIGURE 3:**
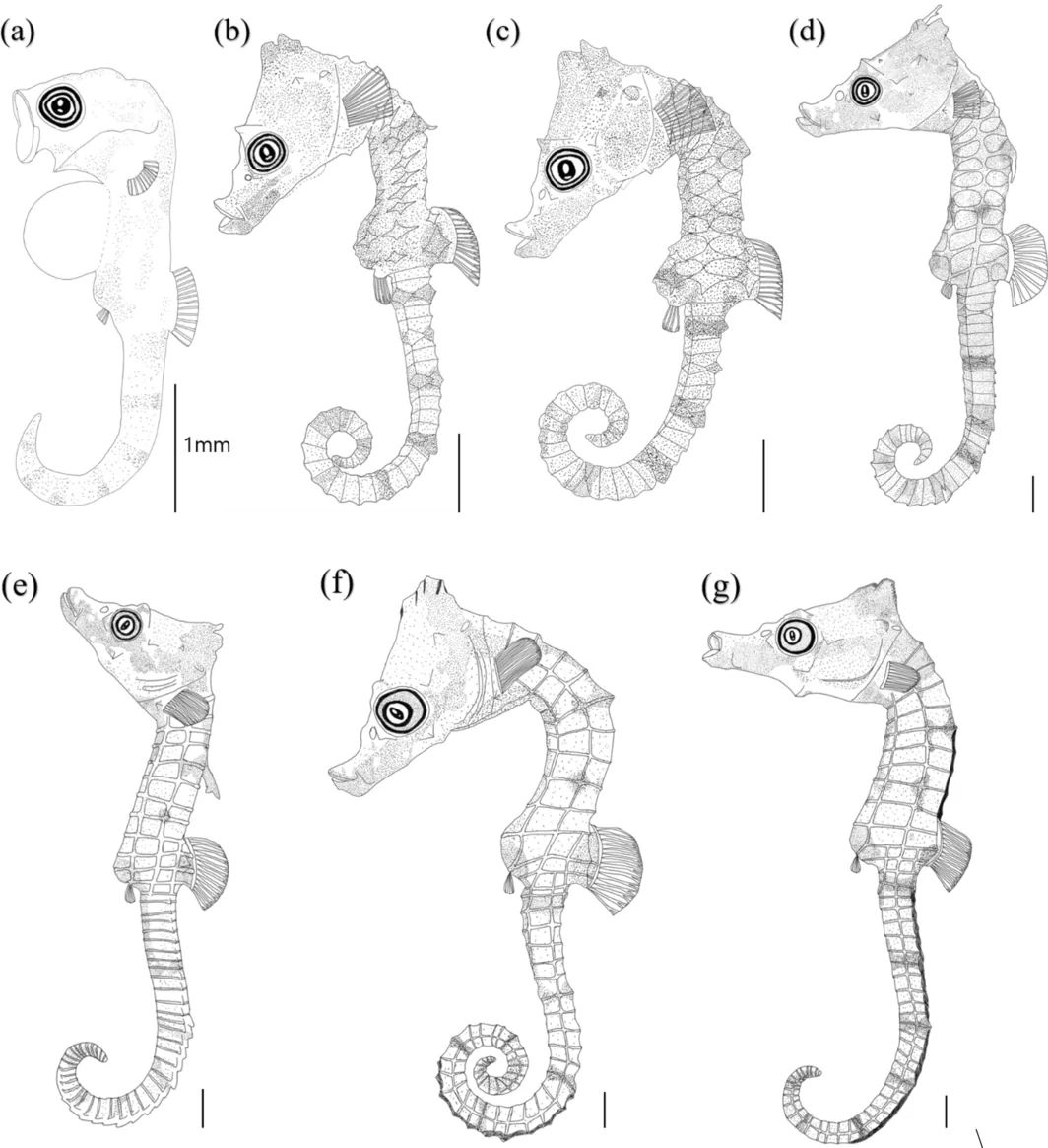
Morphological development of the *Hippocampus haema* juvenile. (a) Prematurely released, 5.93–8.94 mm SL (mean = 6.93 mm, *n* = 5) (PKUI829); (b) day of birth, 11.53–12.39 mm SL (mean = 11.84 mm, *n* = 5) (PKUI830); (c) 4 days after release, 14.15–15.36 mm SL (mean = 14.943 mm, *n* = 5) (PKUI831); (d) 8 days after their release, 16.99–18.63 mm SL (mean = 17.59 mm, *n* = 5) (PKUI833); (e) 15 days after their release, 17.36–17.98 mm SL (mean = 17.95 mm, *n* = 5) (PKUI834); (f) 20 days after their release, 21.17–25.87 mm SL (mean = 22.49 mm, *n* = 5) (PKUI835); and (g) 40 days after their release, 23.78–28.43 mm (mean = 25.64 mm, *n* = 5) (PKUI836). Scale bars = 1 mm. SL, standard length.

Newborns closely resembled miniature adults immediately after release from the pouch. Their SL was 11.53–12.39 mm (mean = 11.84 mm, *n* = 5), and the abdominal yolk was no longer present. The head was angled ventrally, giving the typical horse‐like profile. The tail was already bent and functional for grasping objects (Figure S1). Eye spines (frontal crests) and a nose spine were present above the eyes and nostrils, respectively. The pterotic crest and gill opening were also visible behind the eyes (Figure 3b). A pair of coronets protruded from the midcranial region in a heart‐shaped form, with an anterior coronet spine positioned in front (Figure 4a). Alizarin Red S staining showed that most skeletal elements of the snout were already ossified, and the lower jaw projected beyond the upper jaw (Figure 5a,d). The bony plates of the trunk were ossified but had not yet fully connected, leaving the body rings partly separated and slightly protruding (Figure 5b). Thus, the trunk plates were present, but their incomplete connection made them less clearly defined than the caudal plates. In contrast, the bony plates of the tail were well developed and connected, forming spines on every second or third ring (Figure 5c). All vertebrae and neural arches were clearly formed, and the fin rays and associated supporting elements were nearly fully ossified (Figure 5b). Numerous small black melanophores were distributed from the head to the tail, especially on the abdomen and dorsum. Pigmentation was particularly intense on the snout, anal region, coronet and fin rays, and rows of melanophores were also present on some protruding trunk and tail rings (Figure 3b).

**FIGURE 4:**
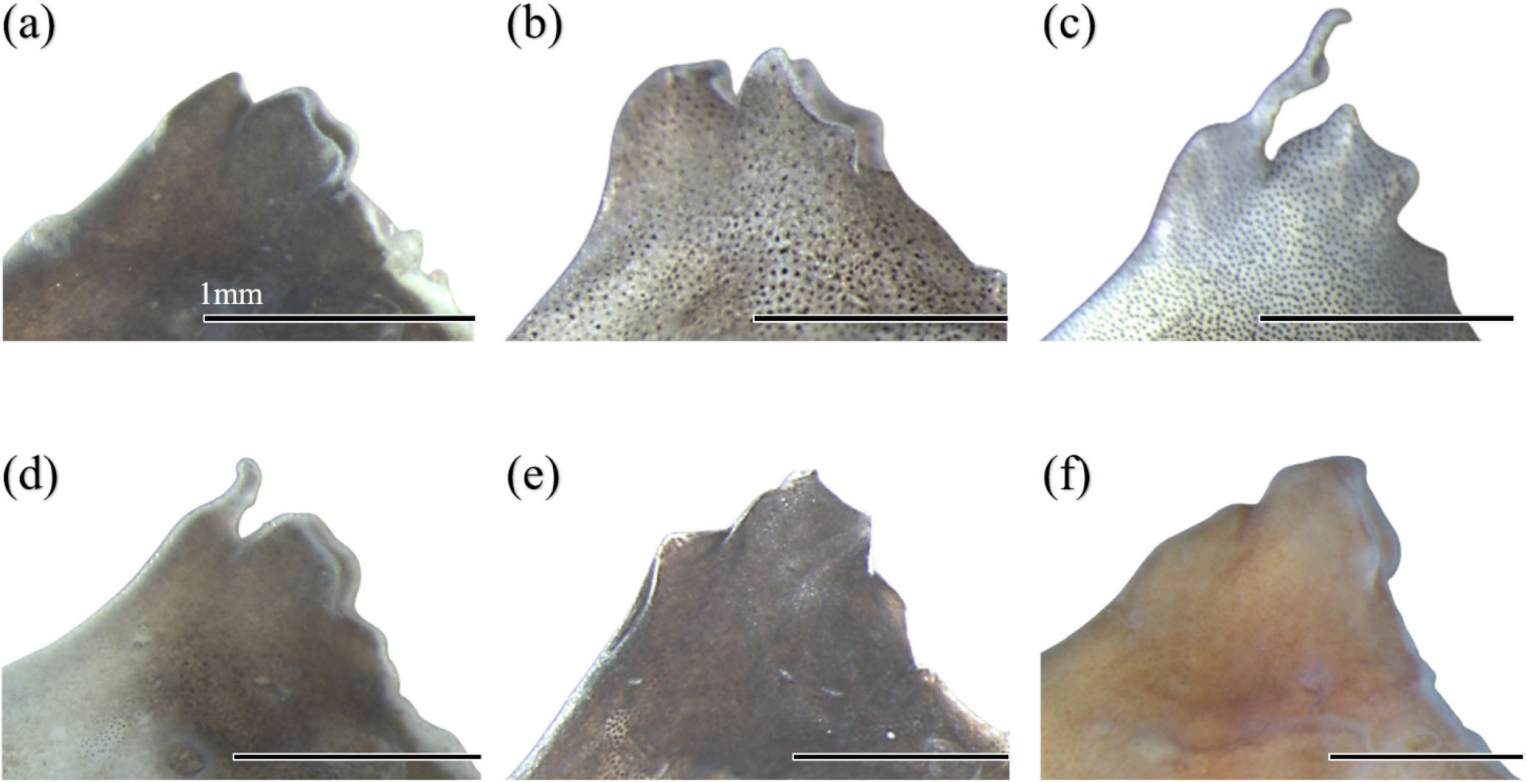
Coronet development of *Hippocampus haema*. (a) day of birth, (b) 4 days after release, (c) 8 days after release, (d) 15 days after release, (e) 20 days after release and (f) 40 days after release. Scale bars = 1 mm.

**FIGURE 5:**
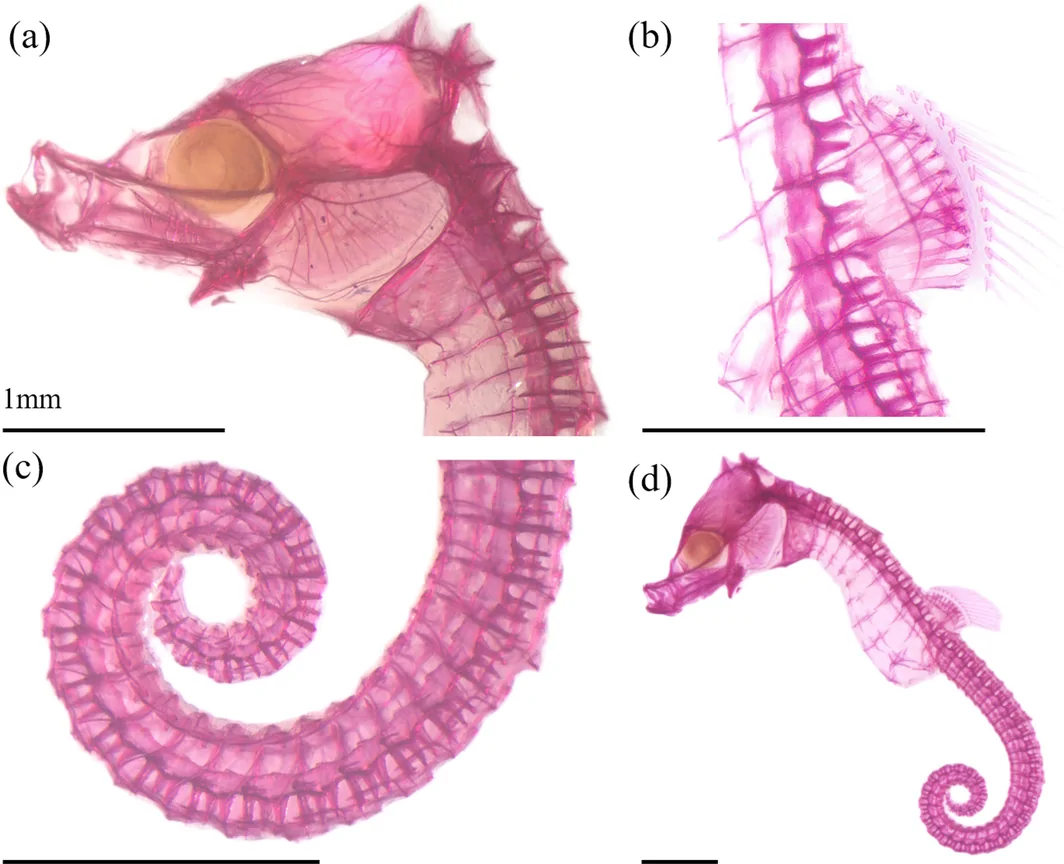
Cleared and stained newborn *Hippocampus haema*. (a) Lateral view of the head showing largely developed cranial skeletal elements at birth. (b) Enlarged view of the trunk region showing ossified but incompletely connected trunk bony plates and dorsal fin skeletal elements. (c) Enlarged view of the tail showing well‐developed caudal skeletal elements. (d) Whole body lateral view of the newborn individual. Scale bars = 1 mm.

Four days after release, the SL of the seahorses was 14.15–15.36 mm (mean = 14.94 mm, *n* = 5). The edges of adjacent bony plates came into contact. The eye spines and bony plate spines were more elongated, and the melanophores became more distinct (Figure 3c). The front spines of the coronet faced rearward and protruded upward (Figure 4b).

Eight days after release, the SL of the seahorses was 16.99–18.62 mm (mean = 17.59 mm, *n* = 5). Skin filaments had grown on the spine of the seventh body ring and the anterior coronet spine (Figure 4c). The bony rings were formed after being linked to the end of each bony plate. The size of the coronet spine had increased, and spines below the eyes appeared. Melanophores on the nose and under the eyes had disappeared. The posterior coronet spine appeared behind the coronet (Figure 3d).

Fifteen days after release, the SL of the seahorses was 17.36–17.98 mm (mean = 17.95 mm, n = 5). The skin filaments on the anterior coronet spine had decreased in length, but the skin filaments on the body ring spine had increased in length (Figure 4d). Additionally, the tail ring spines appeared sharper. The density of the melanophores had increased on the cheeks and nape, while the melanophores on the body and tail had disappeared except on the spine and abdominal region (Figure 3e).

Twenty days after release, the SL of the seahorses was 21.17–25.87 mm (mean = 22.49 mm, *n* = 5). The height of the coronet had increased and was higher than that of the anterior coronet spine (Figure 4e). In addition, the skin filaments on the anterior coronet spine and the body ring spine had disappeared completely (Figure 3f).

Forty days after release, the SL of the seahorses was 23.78–28.43 mm (mean = 25.64 mm, *n* = 5). The anterior coronet spine had extended backwards, and the coronet had extended upward, becoming closer to each other (Figure 4f). Head spines emerged, including the eye and nose spines (Figure 3g).

### 3.3 Growth

Growth in SL was best described by a power function, *y* = 11.335𝑥^0.208^, with a strong model fit (*R* ^2^ = 0.940; Figure 6). Growth was rapid during the early developmental stages but gradually slowed over time, resulting in a progressively flatter curve under the present laboratory conditions.

**FIGURE 6:**
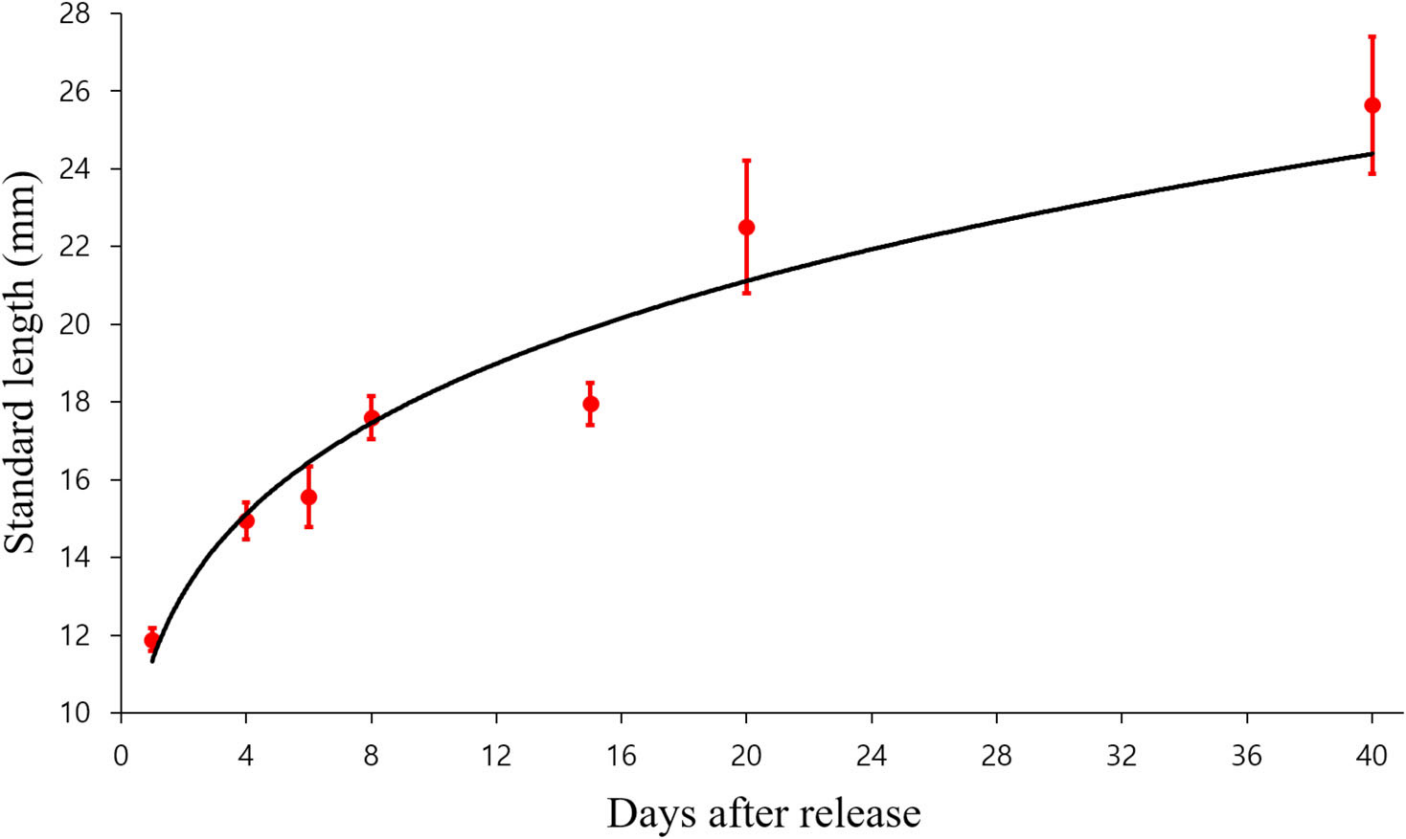
Growth rate of *Hippocampus haema* after release (mm). Error bars represent the standard deviation from the mean.

Morphometric relationships between the SL and measured traits were evaluated by comparing linear and power function models (Table S2). All measured traits increased with SL. The fitted linear and power function curves showed broadly similar trajectories for most traits, and power function models were not clearly better supported than linear models for any measured trait. Linear and power models showed similar support for head length, trunk length, tail length and eye diameter, whereas linear models were better supported for snout depth and snout length based on AICc.

Snout depth was the only trait showing a significant breakpoint (Table S2). Davies' test detected a significant breakpoint for snout depth relative to SL (*p* < 0.001), with the breakpoint occurring at 21.274 mm SL. Before the breakpoint, snout depth increased gradually with SL, with a linear slope of 0.0134. After the breakpoint, the slope increased to 0.0351, indicating an approximately 2.6‐fold steeper increase in snout depth after this point (Figure 7). No significant breakpoint was detected for head length, trunk length, tail length, snout length or eye diameter (Table S2).

**FIGURE 7:**
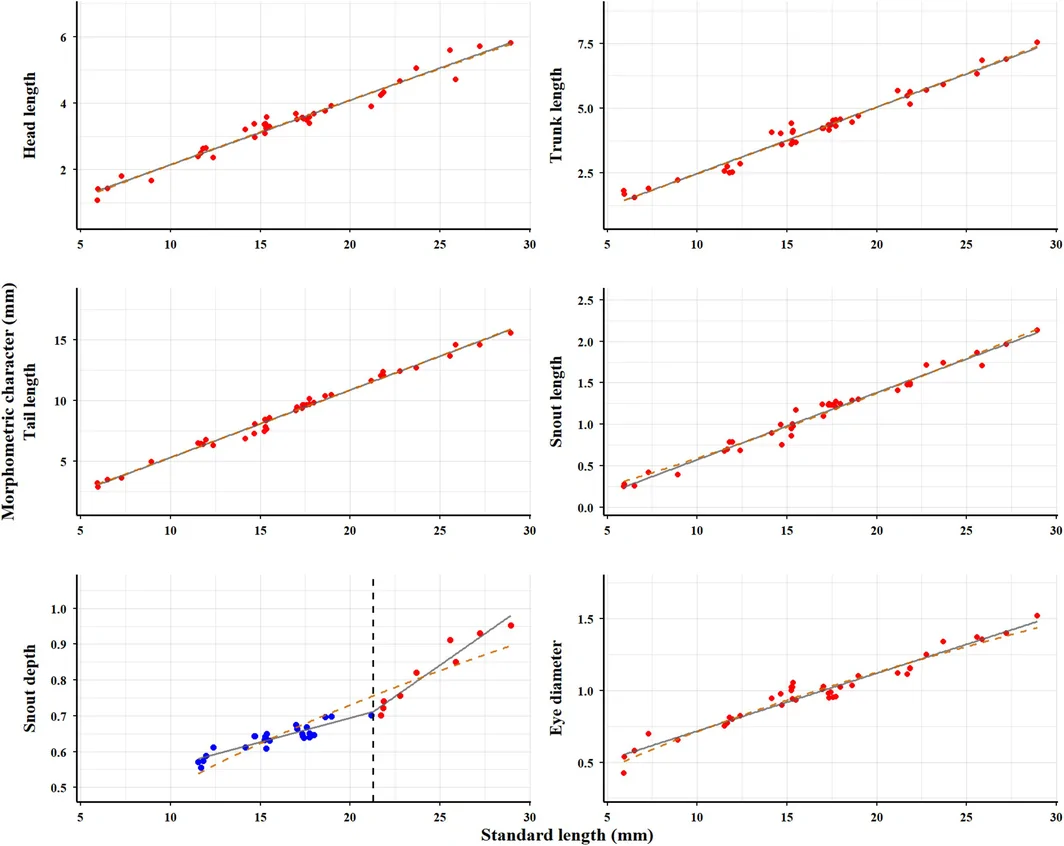
Allometric growth relationships between measured morphometric characters and standard length (SL) of *Hippocampus haema*. Red points indicate observed measurements, and blue points indicate observed measurements below the estimated breakpoint in the snout depth. Black solid lines represent linear models, and orange dashed curves represent power function models. For head length, the linear equation was *y* = 0.207 + 0.194x (*R* ^2^ = 0.967), and the power function equation was *y* = 0.251x^0.932^ (*R* ^2^ = 0.967). For Trunk length, the linear equation was *y* = −0.077 + 0.256x (*R* ^2^ = 0.972), and the power function equation was *y* = 0.230x^1.031^ (*R* ^2^ = 0.973). For Tail length, the linear equation was *y* = −0.248 + 0.557x (*R* ^2^ = 0.990), and the power function equation was *y* = 0.501x^1.027^ (*R* ^2^ = 0.989). For Snout length, the linear equation was *y* = −0.235 + 0.081x (*R* ^2^ = 0.976), and the power function equation was *y* = 0.036x^1.212^ (*R* ^2^ = 0.973). For Snout depth, segmented linear regression showed two growth phases: Y = 0.425 + 0.013x before the breakpoint (*R* ^2^ = 0.808) and y = −0.035 + 0.035x after the breakpoint (*R* ^2^ = 0.928), while the power function equation was *y* = 0.140x^0.552^ (*R* ^2^ = 0.877). For eye diameter, the linear equation was y = 0.316 + 0.040x (*R* ^2^ = 0.945), and the power function equation was *y* = 0.157x^0.657^ (*R* ^2^ = 0.943). The vertical dashed line in the Snout depth panel represents the estimated breakpoint at 21.3 mm SL.

## 4 DISCUSSION

This study identified three major ontogenetic features of *H. haema*. First, newborns possessed a highly developed skeletal framework at birth, particularly in the caudal skeleton, and were able to coil the prehensile tail around a substrate immediately after release. These observations indicate that the major skeletal elements required for tail attachment are already established at birth and that *H. haema* may undergo a very short or indistinct planktonic phase under the observed conditions. Second, coronet morphology showed marked changes, with skin filaments appearing and disappearing across developmental stages, and the anterior coronet spine progressively fusing with the coronet during growth. Third, morphometric model comparison identified a distinct ontogenetic shift in snout development, with snout depth showing a significant breakpoint at 21.3 mm SL, suggesting accelerated development of feeding‐related morphology during early juvenile growth.

Overall, the courtship and mating behaviour of *H. haema* followed the stereotyped sequence described for other *Hippocampus* species (Masonjones & Lewis, 1996; Woods, 2000). Specifically, behaviours such as male brood pouch ’pumping’ and mutual vertical rising aligned with the ethological sequences documented in *H. abdominalis* and *H. zosterae* (Masonjones & Lewis, 1996; Mattle & Wilson, 2009). However, in terms of sexual colour expression, *H. haema* differed from patterns reported in several other *Hippocampus* species. In many seahorses, courtship is accompanied by conspicuous brightening or overall paling in both sexes, and males often show particularly pronounced changes, including increased pouch/body brightness (Mederos et al., 2022; Oliveira et al., 2010; Woods, 2000). By contrast, males of *H. haema* tended to darken or develop an orange hue during courtship (Figure 2), suggesting species‐specific variation in the direction and function of visual signalling during reproduction.

However, reproductive output and the developmental condition of newborns differed more clearly among the compared seahorse species. Newborn *H. haema* were released in a relatively advanced skeletal condition, with most skeletal elements already ossified except for the trunk bony plates (Figure 5). In syngnathid fishes, embryos develop within a male brooding structure and are released after hatching (Kuiter, 2009). Among them, seahorses with a closed brood pouch generally release offspring at a more advanced developmental stage than species with open or semi‐open brooding structures (Sommer et al., 2012). This difference in brooding morphology is also reflected in skeletal development. For example, *H. erectus*, which has a closed brood pouch, is released with ossified cranial and jaw‐related elements, whereas the pipefishes *Nerophis ophidion* and *Syngnathus typhle*, which have open or semi‐open brooding structures, are released with most skeletal elements still cartilaginous or not yet ossified (Schneider et al., 2023). Nevertheless, even in seahorses that are released at a relatively advanced developmental stage, many skeletal elements outside the branchiocranium, such as bony plates and vertebrae, may still be absent, cartilaginous, or only beginning to ossify at birth (Franz‐Odendaal & Adriaens, 2014; Schneider et al., 2023; Shi et al., 2023). To the best of our knowledge, fully ossified caudal bony plates at release have not yet been reported in other seahorse species. This is functionally important because the caudal bony plates are closely associated with the prehensile function of the tail (Neutens et al., 2014, 2017; Porter et al., 2015). In *H. hippocampus*, Novelli et al. (2017) reported that bony plate ossification begins in the trunk region, whereas the caudal bony plates were not ossified until 14 days after birth. They also observed that juveniles were able to bend the tail around a substrate at this stage, suggesting that caudal plate ossification may represent an important developmental cue for the transition from a planktonic to a benthic life stage (Novelli et al., 2017). In contrast, newborn *H. haema* exhibited fully ossified caudal bony plates at release, which is consistent with our observation that juveniles were able to grasp substrates with the tail immediately after birth (Figures 5c and S1). Therefore, the relatively advanced ossification of newborn *H. haema* suggests that this species is released with morphological features that enable immediate substrate attachment, potentially shortening or obscuring the planktonic phase observed in many other syngnathids (Table 2).

**TABLE 2:** Adult size, size at birth and maximum brood size and planktonic phase across seahorses.

Species	Standard length of adult, cm (max)	Standard length at birth, mm (mean)	Number of offspring (max)	Planktonic phase
*Hippocampus haemaᵃ*	7.76	11.82	54	No
*Hippocampus haemaᵇ*	8.73	13.6	38.8	No
*Hippocampus abdominalis*	35	16.2	1116	Yes
*Hippocampus barbouri*	15	5	250	Not reported
*Hippocampus bargibanti*	2.4	2	34	Inferred
*Hippocampus breviceps*	10	8.9	100	Inferred
*Hippocampus capensis*	12	11.1	120	Yes
*Hippocampus comes*	18.7	9.5	350	Yes
*Hippocampus coronatusᶜ*	12.7	9	Several hundred	Not reported
*Hippocampus erectus*	19	11	1552	Inferred
*Hippocampus fuscus*	14.4	7.5	150	Uncertain
*Hippocampus guttulatus*	18	11.8	581	Yes
*Hippocampus hippocampus*	15	9.3	865	Inferred
*Hippocampus ingens*	31	8.5	2000	Not reported
*Hippocampus kuda*	17	7	1405	Inferred
*Hippocampus reidi*	17.5	6.7	1572	Inferred
*Hippocampus subelongatus*	20	11.3	700	Inferred
*Hippocampus trimaculatus*	17	6.1	1783	Not reported
*Hippocampus whitei*	13	8.5	250	Not reported
*Hippocampus zosterae*	2.5	7.9	55	Not reported

*Note*: Values are compiled from Foster and Vincent (2004), unless otherwise indicated, with exceptions marked by superscripts and cited in footnotes (e.g., ᵃPresent study, ᵇChoi et al. (2006), ᶜMasuda et al. (1984)). In the ‘Planktonic phase’ column, ‘Yes’ indicates that a planktonic phase has been reported, ‘No’ indicates that no evident planktonic phase has been observed, ‘Inferred’ indicates that the presence of a planktonic phase was inferred rather than directly observed and ‘Not Reported’ indicates that no information was available in the cited source.

This advanced phenotype was accompanied by unusually large newborn size. Despite having a small maximum adult size (7.76–8.73 cm), *H. haema* produced larger newborns than similarly sized species such as *H. breviceps*, *H. capensis* and *H. whitei*, and even exceeded the reported newborn sizes of substantially larger species, including *H. comes*, *H. erectus*, *H. ingens* and *H. subelongatus* (Table 2). In contrast, brood output in *H. haema* was low (maximum 54 offspring). A similarly small brood size was also observed in the wild, with a mean of 26.8 ± 9.2 offspring (Park & Kwak, 2015). Their brood output was far below the hundreds to thousands reported for many seahorses, and comparable to values reported for small species, such as *H. bargibanti* (34) and *H. zosterae* (55) (Table 2). In seahorses, brood size and offspring size are often constrained by the carrying capacity of the male brood pouch, creating a trade‐off whereby smaller broods can be associated with larger, more developed newborns (Faleiro et al., 2016). The combination of small broods and large, developmentally advanced newborns in *H. haema* is therefore consistent with a strategy of increased per‐offspring investment. Nonetheless, stronger inference will require more standardised interspecific comparisons that account for differences among species in gestation duration, habitat‐specific food availability and the magnitude of intraspecific size variation.

Juvenile growth in *H. haema* showed a non‐linear trajectory, with a rapid increase in SL shortly after release, followed by a more gradual increase during later development (Figure 6). This overall trajectory was broadly similar to that reported for *H. haema* by Choi et al. (2006), although juveniles in the present study were generally slightly larger at comparable ages. By contrast, some other seahorses show more distinct stage‐dependent changes in post‐release growth rate. For example, *H. abdominalis*, *H. kuda*, *H. hippocampus* and *H. trimaculatus* showed a relatively slow increase in SL during the early period, followed by a marked increase during later development (Choo & Liew, 2006; Murugan et al., 2009; Novelli et al., 2017; Shi et al., 2023). Similarly, early growth in *H. reidi* has been divided into three developmental stages, with relatively rapid growth during the first stage, slower growth during the second stage and renewed acceleration during the third stage (Peña et al., 2025). In that species, multiple inflection points in allometric growth were interpreted as developmental markers of changes in growth rate and body shape, coinciding with the transition from an actively swimming pelagic stage to a more benthic stage characterised by reduced swimming activity, substrate attachment and ambush feeding (Peña et al., 2025). Therefore, the absence of a distinct stage‐dependent shift in the standard‐length growth of *H. haema* may reflect the fact that some morphological and behavioural traits associated with substrate attachment are already established at release. In particular, the immediate use of the prehensile tail and the relatively advanced skeletal condition of newborns suggest that the transition from a pelagic to a substrate‐associated stage may be shortened or indistinct in this species. However, to test this interpretation more explicitly, detailed comparative data on SL and morphometric traits are needed from other syngnathid species that lack a planktonic larval phase.

Previous studies of juvenile seahorses have evaluated the growth of individual body parts relative to SL using power function models, with allometric or isometric growth inferred from whether the scaling coefficient differed from 1 (Choo & Liew, 2006; Peña et al., 2025; Shi et al., 2023). In the present study, however, morphometric model comparison did not provide strong support for power function models over linear models for the measured traits. Therefore, most size‐related changes in body measurements are interpreted here as morphometric relationships with SL rather than as clear evidence of continuous allometric growth. This interpretation is consistent with Packard's (2024) recommendation that allometric patterns should be evaluated by comparing alternative candidate models fitted to the observed data rather than assumed from power function fits alone.

Nevertheless, snout depth showed clear evidence of a discrete ontogenetic shift, as segmented regression detected a significant breakpoint at approximately 21.3 mm SL (Table S2). Before this breakpoint, snout depth increased gradually with SL, whereas the post‐breakpoint slope was approximately 2.6 times steeper (Figure 7). Comparable developmental changes in snout traits have also been reported in other juvenile seahorses. In *H. kuda*, morphometric ratios of several body parts, including snout length and snout depth, changed around a transition point of approximately 25 mm SL, which was interpreted as potentially related to a dietary shift from copepods to larger mysids (Choo & Liew, 2006). Similarly, Peña et al. (2025) reported stage‐dependent changes in growth coefficients and inflection points for snout length and snout height in *H. reidi*, suggesting that snout traits change during early development in association with feeding and body shape transitions. Therefore, the accelerated increase in snout depth after the breakpoint in *H. haema* may be associated with a feeding‐related morphological transition during juvenile growth.

This interpretation is biologically plausible because, in syngnathids, interspecific differences in snout length and depth can reflect the type and size of prey consumed (Kendrick & Hyndes, 2005). Even within a species, developmental transitions in head and snout morphology can occur in association with ontogenetic shifts in prey preference between juveniles and adults (Leysen, Roos, & Adriaens, 2011). In *H. haema*, small individuals (<30 mm SL) have been reported to feed predominantly on copepods (>90%), whereas individuals ≥30 mm SL shift to a diet dominated by gammarid amphipods (>60%) (Huh et al., 2014). In this context, the slow increase in snout depth prior to the breakpoint may reflect morphological constraints related to maintaining suction feeding performance when capturing small, fast prey such as copepods because snout dimension could influence suction feeding hydrodynamics and performance (Roos et al., 2011; Van Wassenbergh et al., 2006). By contrast, the pronounced post‐breakpoint increase may be associated with expanding oral capacity and prey handling ability to capture and process larger, bulkier prey (gammarids). Nevertheless, confirming this interpretation will require size‐classified quantitative diet data (or prey size distributions and stomach contents) collected from the same developmental window to directly test the coupling between snout morphology and diet shift.

The *H. coronatus* complex (*H. haema*, *H. coronatus* and *H. sindonis*) can be distinguished from many other *Hippocampus* species by having 10 trunk rings (vs. 11 in most seahorses). However, within the complex, species identification relies primarily on coronet characters, and the diagnostic features differ among species. *H. sindonis* is separated from *H. haema* and *H. coronatus* by coronet tip number (typically five in *H. sindonis* vs. four in *H. haema* and *H. coronatus*) (Han et al., 2017), whereas adult discrimination between *H. haema* and *H. coronatus* commonly depends on the coronet height. A key limitation highlighted by our data is that these coronet characters are poorly expressed in early life stages: newborns in the present study had little to no coronet development, and coronet morphology changed substantially through juvenile growth (Figure 4). Consequently, adult‐based identification keys are likely to be unreliable for juveniles, and distinguishing species within the complex during early ontogeny may be intrinsically difficult. Skin filaments, which are frequently used in seahorse identification, first appeared at 8 days post‐release (Figure 4c), began to regress by ~15 days (Figure 4d), and were absent by ~20 days (Figure 4e). Because filament expression can be environmentally plastic, appearing or disappearing depending on habitat structure or rearing conditions, it should be treated as an auxiliary character rather than a fixed diagnostic trait for *H. haema* (Curtis, 2006; Han et al., 2017). Accordingly, juvenile identification should avoid reliance on single, potentially plastic traits and instead incorporate multiple characters together with the developmental stage.

Importantly, coronet architecture followed a relatively consistent developmental trajectory. By ~20 days post‐release, the coronet became taller than the anterior coronet spine (Figure 4e), and by ~40 days the anterior coronet spine and coronet effectively fused into a single, continuous structure (Figure 4f). This trajectory potentially contrasts with *H. sindonis*, in which the anterior coronet spine has been described as remaining comparable in size to the main coronet spine, suggesting that coronet configuration could aid separation of juveniles from around 20 days onward. However, because juvenile stage coronet development data for *H. sindonis* and *H. coronatus* remain limited, future work should obtain specimens across defined ontogenetic stages and quantitatively compare coronet traits, including coronet height and the number of coronet tips. Such comparative datasets are expected to help establish more reliable species identification criteria, even at juvenile stages.

In the present study, newborn *H. haema* bent their tails and attached immediately to nearby structures after release. This observation contrasts with reports for other *Hippocampus* species, such as *H. kuda* and *H. hippocampus*, whose newborns have been described as remaining planktonic for approximately two weeks before showing regular substrate attachment (Choo & Liew, 2006; Novelli et al., 2017). The immediate attachment observed in newborn *H. haema* indicates that they are functionally capable of substrate attachment at birth and suggests a possible early benthic tendency under the observed conditions. Early settlement could limit demographic rescue and recolonisation among neighbouring habitats, thereby reducing population connectivity and increasing the likelihood of population subdivision even at fine geographic scales (Hoffman et al., 2005; Planes et al., 2001). Strong genetic differentiation has also been reported for the dwarf seahorse *H. zosterae*, which is characterised by early settlement and limited dispersal, supporting the idea that reduced early dispersal can reinforce population structuring (Fedrizzi et al., 2015).

From a conservation perspective, reduced connectivity implies constrained immigration and recolonisation following disturbance, such that habitat degradation can translate more directly into local population declines and an elevated risk of local extinction (Fahrig, 2002; Hanski, 1998). These concerns are further underscored by the recent listing of *H. haema* as Vulnerable on the IUCN Red List (IUCN, 2025). Moreover, some predictive studies have suggested that the inherently limited dispersal capacity of seahorses may restrict range shifts even if climate change alters the distribution of suitable habitats (Peiffer et al., 2025); in particular, small and isolated populations may be more vulnerable to loss of genetic diversity (e.g., bottlenecks and inbreeding depression) and local mortality under environmental stress (Coleman et al., 2018; Keller & Waller, 2002). Therefore, conservation of *H. haema* should prioritise protecting and restoring core habitats that function as spawning and nursery areas (e.g., eelgrass beds) (Cho et al., 2022; M. J. Kim et al., 2018), together with designing protected area networks that maintain fine‐scale habitat connectivity and minimise fragmentation (Balbar & Metaxas, 2019; Peiffer et al., 2024). To empirically test these hypothesised implications, population genetic studies are also needed to quantify effective population size, genetic diversity and levels of genetic differentiation between regional populations, thereby providing an evidence base for defining management units and setting conservation priorities.

## AUTHOR CONTRIBUTIONS

J.J.L: Conceptualisation; Investigation (rearing and observation); Formal analysis (allometry); Visualisation (illustrations and graphs); Writing—original draft. J.K.K: Conceptualisation; Supervision; Writing—review & editing.

## FUNDING INFORMATION

This work was supported by the management of the Marine and Fishery Bio‐resources Centre (2026) funded by the National Marine Biodiversity Institute of Korea (MABIK).

## CONFLICT OF INTEREST STATEMENT

The authors declare that they have no conflict of interest.

## Supporting information


**Figure S1.** Photograph of newborn *Hippocampus haema*. The newborn individual coiled its prehensile tail around an object and attached to it immediately after release.
**Table S1.** Individual morphometric measurements (mm) of Hippocampus haema across developmental stages. Note: NA indicates that snout depth could not be measured in specimens collected five days before release.
**Table S2.** Model comparison and breakpoint analysis for morphometric traits of *Hippocampus haema*. Linear, power function and segmented regression models were compared using AICc and *R* ^2^ values. Breakpoints were tested using Davies' test. A dash indicates that no statistically significant breakpoint was detected. The asterisk indicates statistical significance at *p* < 0.05.

## Data Availability

The data that supports the findings of this study are available in the Supporting Information of this article.

## References

[jfb70593-bib-0001] Balbar, A. C. , & Metaxas, A. (2019). The current application of ecological connectivity in the design of marine protected areas. Global Ecology and Conservation, 17, e00569. 10.1016/j.gecco.2019.e00569

[jfb70593-bib-0002] Burnham, K. P. , & Anderson, D. R. (2002). Model selection and multimodel inference: A practical information‐theoretic approach. Springer.

[jfb70593-bib-0003] Chambers, R. C. , & Trippel, E. A. (1997). Early life history and recruitment: Legacy and challenges. In Early life history and recruitment in fish populations (pp. 515–549). Springer.

[jfb70593-bib-0004] Cho, H.‐G. , Ahn, J.‐K. , & Kim, H.‐S. (2022). Habitat characteristics and spawning ecology of hippocampus haema (Pisces: Syngnathidae) inhabiting the Soando (island). Korean Journal of Environment and Ecology, 36(6), 615–626.

[jfb70593-bib-0006] Choi, Y.‐U. , Rho, S. , Jung, M.‐M. , Lee, Y.‐D. , & Noh, G.‐A. (2006). Parturition and early growth of crowned seahorse, Hippocampus coronatus in Korea. Journal of Aquaculture, 19(2), 109–118.

[jfb70593-bib-0005] Choi, Y.‐U. , Rho, S. , Park, H.‐S. , & Kang, D.‐H. (2012). Population characteristics of two seahorses, Hippocampus coronatus and Hippocampus mohnikei, around seagrass beds in the southern coastal waters of Korea. Ichthyological Research, 59(3), 235–241.

[jfb70593-bib-0007] Choo, C. K. , & Liew, H. C. (2006). Morphological development and allometric growth patterns in the juvenile seahorse Hippocampus kuda Bleeker. Journal of Fish Biology, 69(2), 426–445. 10.1111/j.1095-8649.2006.01105.x

[jfb70593-bib-0008] CITES Secretariat . (2026). Convention on International Trade in Endangered Species of Wild Fauna and Flora . https://cites.org 10.1159/000459796 712806

[jfb70593-bib-0009] Coleman, R. A. , Gauffre, B. , Pavlova, A. , Beheregaray, L. B. , Kearns, J. , Lyon, J. , Sasaki, M. , Leblois, R. , Sgro, C. , & Sunnucks, P. (2018). Artificial barriers prevent genetic recovery of small isolated populations of a low‐mobility freshwater fish. Heredity, 120(6), 515–532. 10.1038/s41437-017-0008-3 PMC5943333 29326479

[jfb70593-bib-0010] Curtis, J. M. R. (2006). A case of mistaken identity: Skin filaments are unreliable for identifying Hippocampus guttulatus and Hippocampus hippocampus. Journal of Fish Biology, 69(6), 1855–1859.

[jfb70593-bib-0011] Curtis, J. M. R. , Santos, S. V. , Nadeau, J. L. , Gunn, B. , Bigney Wilner, K. , Balasubramanian, H. , Overington, S. , Lesage, C.‐M. , D'entremont, J. , & Wieckowski, K. (2017). Life history and ecology of the elusive European short‐snouted seahorse Hippocampus hippocampus. Journal of Fish Biology, 91(6), 1603–1622. 10.1111/jfb.13473 29068054

[jfb70593-bib-0012] Curtis, J. M. R. , & Vincent, A. C. (2006). Life history of an unusual marine fish: Survival, growth and movement patterns of Hippocampus guttulatus Cuvier 1829. Journal of Fish Biology, 68(3), 707–733.

[jfb70593-bib-0013] Davies, R. B. (1987). Hypothesis testing when a nuisance parameter is present only under the alternative. Biometrika, 74(1), 33–43.

[jfb70593-bib-0014] Fahrig, L. (2002). Effect of habitat fragmentation on the extinction threshold: A synthesis. Ecological Applications, 12(2), 346–353. 10.1890/1051-0761(2002)012[0346:EOHFOT]2.0.CO;2

[jfb70593-bib-0015] Faleiro, F. , Almeida, A. J. , Ré, P. , & Narciso, L. (2016). Size does matter: An assessment of reproductive potential in seahorses. Animal Reproduction Science, 170, 61–67. 10.1016/j.anireprosci.2016.04.003 27062576

[jfb70593-bib-0016] Fedrizzi, N. , Stiassny, M. L. , Boehm, J. T. , Dougherty, E. R. , Amato, G. , & Mendez, M. (2015). Population genetic structure of the dwarf seahorse (Hippocampus zosterae) in Florida. PLoS One, 10(7), e0132308. 10.1371/journal.pone.0132308 PMC4511636 26200110

[jfb70593-bib-0017] Foster, S. , & Vincent, A. C. (2004). Life history and ecology of seahorses: Implications for conservation and management. Journal of Fish Biology, 65(1), 1–61.

[jfb70593-bib-0018] Franz‐Odendaal, T. A. , & Adriaens, D. (2014). Comparative developmental osteology of the seahorse skeleton reveals heterochrony amongst hippocampus sp. and progressive caudal fin loss. EvoDevo, 5(1), 45. 10.1186/2041-9139-5-45 PMC4407769 25908960

[jfb70593-bib-0019] Froese, R. , & Pauly, D. (2026). FAMILY Details for Syngnathidae—Pipefishes and seahorses. https://www.fishbase.se/summary/FamilySummary.php?ID=258

[jfb70593-bib-0020] Fuiman, L. A. (1983). Growth gradients in fish larvae. Journal of Fish Biology, 23(1), 117–123.

[jfb70593-bib-0021] Hamilton, H. , Saarman, N. , Short, G. , Sellas, A. B. , Moore, B. , Hoang, T. , Grace, C. L. , Gomon, M. , Crow, K. , & Simison, W. B. (2017). Molecular phylogeny and patterns of diversification in syngnathid fishes. Molecular Phylogenetics and Evolution, 107, 388–403. 10.1016/j.ympev.2016.10.003 27989632

[jfb70593-bib-0022] Han, S.‐Y. , Kim, J.‐K. , Kai, Y. , & Senou, H. (2017). Seahorses of the Hippocampus coronatus complex: Taxonomic revision, and description of hippocampus haema, a new species from Korea and Japan (Teleostei, Syngnathidae). ZooKeys, 712, 113–139. 10.3897/zookeys.712.14955 PMC5704180 29187790

[jfb70593-bib-0023] Hanski, I. (1998). Metapopulation dynamics. Nature, 396(6706), 41–49. 10.1038/23876

[jfb70593-bib-0024] Harasti, D. , Martin‐Smith, K. , & Gladstone, W. (2012). Population dynamics and life history of a geographically restricted seahorse, Hippocampus whitei. Journal of Fish Biology, 81(4), 1297–1314. 10.1111/j.1095-8649.2012.03406.x 22957871

[jfb70593-bib-0025] Hobbs, J. A. , Lewis, L. S. , Willmes, M. , Denney, C. , & Bush, E. (2019). Complex life histories discovered in a critically endangered fish. Scientific Reports, 9(1), 16772. 10.1038/s41598-019-52273-8 PMC6856525 31727901

[jfb70593-bib-0026] Hoffman, E. A. , Kolm, N. , Berglund, A. , Arguello, J. R. , & Jones, A. G. (2005). Genetic structure in the coral‐reef‐associated Banggai cardinalfish, Pterapogon kauderni. Molecular Ecology, 14(5), 1367–1375. 10.1111/j.1365-294X.2005.02538.x 15813777

[jfb70593-bib-0027] Houde, A. E. (1987). Mate choice based upon naturally occurring color‐pattern variation in a guppy population. Evolution, 41(1), 1–10. 10.1111/j.1558-5646.1987.tb05766.x 28563755

[jfb70593-bib-0028] Huh, S.‐H. , Park, J. M. , Kwak, S. N. , & Seong, B. J. (2014). Abundances and feeding habits of Hippocampus coronatus in an eelgrass (Zostera marina) bed of Dongdae Bay, Korea. Journal of the Korean Society of Fisheries and Ocean Technology, 50(2), 115–123. 10.3796/KSFT.2014.50.2.115

[jfb70593-bib-0029] IUCN . (2025). Guidelines for Using the IUCN Red List Categories and Criteria . https://www.iucnredlist.org

[jfb70593-bib-0030] Kanou, K. , & Kohno, H. (2001). Early life history of a seahorse, Hippocampus mohnikei, in Tokyo Bay, Japan. Ichthyological Research, 48(4), 361–368. 10.1007/s10228-001-8159-9

[jfb70593-bib-0031] Keller, L. F. , & Waller, D. M. (2002). Inbreeding effects in wild populations. Trends in Ecology & Evolution, 17(5), 230–241. 10.1016/S0169-5347(02)02489-8

[jfb70593-bib-0032] Kendrick, A. J. , & Hyndes, G. A. (2005). Variations in the dietary compositions of morphologically diverse syngnathid fishes. Environmental Biology of Fishes, 72(4), 415–427.

[jfb70593-bib-0033] Kim, M. J. , Kim, H. C. , Lee, W. C. , Park, J. M. , Kwak, S. N. , Oh, Y. , Kang, M. G. , & Lee, S. H. (2018). Ecological characteristics of the new recorded seahorse (hippocampus haema) in Geoje‐Hansan Bay, Korea. Journal of Coastal Research, 85(sp1), 351–355.

[jfb70593-bib-0034] Kim, M.‐J. , Kim, H.‐W. , Lee, S.‐R. , Kim, N.‐Y. , Lee, Y.‐J. , Joo, H.‐T. , Kwak, S.‐N. , & Lee, S.‐H. (2022). Feeding strategy of the wild Korean seahorse (hippocampus haema). Journal of Marine Science and Engineering, 10(3), 357.

[jfb70593-bib-0035] Koning, S. , & Hoeksema, B. W. (2021). Diversity of seahorse species (hippocampus spp.) in the international aquarium trade. Diversity, 13(5), 187.

[jfb70593-bib-0036] Kuiter, R. H. (2009). Seahorses and their relatives (p. 208). Aquatic Photographics.

[jfb70593-bib-0037] Leysen, H. , Dumont, E. R. , Brabant, L. , Van Hoorebeke, L. , & Adriaens, D. (2011). Modelling stress in the feeding apparatus of seahorses and pipefishes (Teleostei: Syngnathidae). Biological Journal of the Linnean Society, 104(3), 680–691. 10.1111/j.1095-8312.2011.01733.x

[jfb70593-bib-0038] Leysen, H. , Roos, G. , & Adriaens, D. (2011). Morphological variation in head shape of pipefishes and seahorses in relation to snout length and developmental growth. Journal of Morphology, 272(10), 1259–1270. 10.1002/jmor.10982 21630324

[jfb70593-bib-0039] Lourie, S. A. , Foster, S. J. , Cooper, E. W. , & Vincent, A. C. (2004). A guide to the identification of seahorses (Vol. 114(1), pp. 1–120). Project Seahorse and TRAFFIC North America.

[jfb70593-bib-0040] Lourie, S. A. , Vincent, A. C. , & Hall, H. J. (1999). Seahorses: An identification guide to the world's species and their conservation. (No Title).

[jfb70593-bib-0041] Masonjones, H. D. , & Lewis, S. M. (1996). Courtship behavior in the dwarf seahorse, Hippocampus zosterae. Copeia, 1996, 634–640.

[jfb70593-bib-0070] Masuda, H. , Uyeno, T. , & Yoshino, T. (1984). The fishes of the Japanese Archipelago, K. Amaoka , & C. Araga (Eds.). (Vol. 1, p. 437). Tokai University Press.

[jfb70593-bib-0042] Mattle, B. , & Wilson, A. B. (2009). Body size preferences in the pot‐bellied seahorse Hippocampus abdominalis: Choosy males and indiscriminate females. Behavioral Ecology and Sociobiology, 63(10), 1403–1410. 10.1007/s00265-009-0804-8 PMC2714888 19633734

[jfb70593-bib-0043] Mederos, S. L. , Duarte, R. C. , Mastoras, M. , Dennis, M. Y. , Settles, M. L. , Lau, A. R. , Scott, A. , Woodward, K. , Johnson, C. , & Seelke, A. M. (2022). Effects of pairing on color change and central gene expression in lined seahorses. Genes, Brain, and Behavior, 21(5), e12812. 10.1111/gbb.12812 PMC9744553 35652318

[jfb70593-bib-0044] Miller, D. J. , & Lea, R. N. (1972). Fish Bulletin 157. Guide To The Coastal Marine Fishes of California. https://escholarship.org/uc/item/6s04v367

[jfb70593-bib-0045] Morgan, S. K. , & Vincent, A. C. (2013). Life‐history reference points for management of an exploited tropical seahorse. Marine and Freshwater Research, 64(3), 185–200.

[jfb70593-bib-0046] Muggeo, V. M. (2008). Segmented: An R package to fit regression models with broken‐line relationships. R News, 8(1), 20–25.

[jfb70593-bib-0047] Murugan, A. , Dhanya, S. , Sreepada, R. A. , Rajagopal, S. , & Balasubramanian, T. (2009). Breeding and mass‐scale rearing of three spotted seahorse, *Hippocampus trimaculatus* leach under captive conditions. Aquaculture, 290(1), 87–96. 10.1016/j.aquaculture.2009.01.033

[jfb70593-bib-0048] Neutens, C. , Adriaens, D. , Christiaens, J. , De Kegel, B. , Dierick, M. , Boistel, R. , & Van Hoorebeke, L. (2014). Grasping convergent evolution in syngnathids: A unique tale of tails. Journal of Anatomy, 224(6), 710–723. 10.1111/joa.12181 PMC4025897 24697519

[jfb70593-bib-0049] Neutens, C. , de Dobbelaer, B. , Claes, P. , & Adriaens, D. (2017). Prehensile and non‐prehensile tails among syngnathid fishes: What's the difference? Zoology, 120, 62–72. 10.1016/j.zool.2016.11.002 27894827

[jfb70593-bib-0050] Novelli, B. , Otero‐Ferrer, F. , Socorro, J. A. , Caballero, M. J. , Segade‐Botella, A. , & Molina Domínguez, L. (2017). Development of short‐snouted seahorse (Hippocampus hippocampus, L. 1758): Osteological and morphological aspects. Fish Physiology and Biochemistry, 43(3), 833–848. 10.1007/s10695-017-0339-2 28124205

[jfb70593-bib-0051] Oliveira, T. P. R. , Castro, A. L. C. , & Rosa, I. L. (2010). Novel sex‐related characteristics of the longsnout seahorse Hippocampus reidi Ginsburg, 1933. Neotropical Ichthyology, 8, 373–378. 10.1590/S1679-62252010000200017

[jfb70593-bib-0052] Osse, J. W. M. , & Van den Boogaart, J. G. M. (2004). Allometric growth in fish larvae: Timing and function. In Symposium held at the 26th annual larval fish conference, of the early life history section of the American fisheries society, in Bergen, Norway (pp. 167–194). American Fisheries Society.

[jfb70593-bib-0053] Packard, G. C. (2024). Data transformation and model selection in bivariate allometry. Biology Open, 13(9), bio060587. 10.1242/bio.060587 PMC11427898 39284732

[jfb70593-bib-0054] Park, J. M. , & Kwak, S. N. (2015). Length–weight relationships and reproductive characteristics of the crowned seahorse (Hippocampus coronatus) in eelgrass beds (Zostera marina) of Dongdae Bay, Korea. Marine Biology Research, 11(2), 209–213. 10.1080/17451000.2014.902536

[jfb70593-bib-0055] Payne, M. F. , & Rippingale, R. J. (2000). Rearing west Australian seahorse, *Hippocampus subelongatus*, juveniles on copepod nauplii and enriched *Artemia* . Aquaculture, 188(3), 353–361. 10.1016/S0044-8486(00)00349-5

[jfb70593-bib-0056] Peiffer, F. , Assis, J. , Lima, A. R. A. , Henriques, S. , Pardal, M. A. , Martinho, F. , Gonçalves, J. M. S. , Gonçalves, E. J. , Correia, M. , & Silva, G. J. F. (2025). Projected climate change and limited dispersal potential threaten the seahorse species Hippocampus hippocampus and Hippocampus guttulatus. Marine Biology, 172(9), 150. 10.1007/s00227-025-04717-1

[jfb70593-bib-0057] Peiffer, F. , Lima, A. R. A. , Henriques, S. , Pardal, M. A. , Martinho, F. , Gonçalves, J. M. S. , Gonçalves, E. J. , Correia, M. , & Silva, G. J. F. (2024). Habitat suitability of two flagship species, *Hippocampus hippocampus* and *Hippocampus guttulatus*, in the Atlantic coast of the Iberian Peninsula ‐ implications for conservation. Global Ecology and Conservation, 53, e02993. 10.1016/j.gecco.2024.e02993

[jfb70593-bib-0058] Peña, R. , Mata‐Miranda, R. P. , & Zúñiga‐Villarreal, E. (2025). Allometric growth during the early development of the long‐snout seahorse Hippocampus reidi Ginsburg, 1933. Latin American Journal of Aquatic Research, 53(5), 788–800. 10.3856/vol53-issue5-fulltext-3385

[jfb70593-bib-0059] Planes, S. , Doherty, P. J. , & Bernardi, G. (2001). Strong genetic divergence among populations of a marine fish with limited dispersal, Acanthochromis polyacanthus, within the great barrier reef and the Coral Sea. Evolution, 55(11), 2263–2273. 10.1111/j.0014-3820.2001.tb00741.x 11794786

[jfb70593-bib-0060] Porter, M. M. , Adriaens, D. , Hatton, R. L. , Meyers, M. A. , & McKittrick, J. (2015). Why the seahorse tail is square. Science, 349(6243), aaa6683. 10.1126/science.aaa6683 26138983

[jfb70593-bib-0061] Roos, G. , Van Wassenbergh, S. , Aerts, P. , Herrel, A. , & Adriaens, D. (2011). Effects of snout dimensions on the hydrodynamics of suction feeding in juvenile and adult seahorses. Journal of Theoretical Biology, 269(1), 307–317. 10.1016/j.jtbi.2010.10.023 21029742

[jfb70593-bib-0062] Schneider, R. F. , Woltering, J. M. , Adriaens, D. , & Roth, O. (2023). A comparative analysis of the ontogeny of syngnathids (pipefishes and seahorses) reveals how heterochrony contributed to their diversification. Developmental Dynamics, 252(5), 553–588. 10.1002/dvdy.551 36351887

[jfb70593-bib-0063] Shi, X. , Tang, X. , Zhang, Y. , Wang, W. , Qin, S. , Liu, Q. , & Mei, J. (2023). The early Allometric growth and osteological ontogeny of pot‐bellied seahorse (Hippocampus abdominalis, L. 1827) under mass‐scale captive breeding conditions in North China. Fishes, 8(12), 604. 10.3390/fishes8120604

[jfb70593-bib-0064] Sommer, S. , Whittington, C. M. , & Wilson, A. B. (2012). Standardised classification of pre‐release development in male‐brooding pipefish, seahorses, and seadragons (family Syngnathidae). BMC Developmental Biology, 12(1), 39. 10.1186/1471-213X-12-39 PMC3541971 23273265

[jfb70593-bib-0065] Taylor, W. R. , & Van Dyke, G. C. (1985). Revised procedures for staining and clearing small fishes and other vertebrates for bone and cartilage study. Cybium, 9, 107–109.

[jfb70593-bib-0066] Van Wassenbergh, S. , Aerts, P. , & Herrel, A. (2006). Hydrodynamic modelling of aquatic suction performance and intra‐oral pressures: Limitations for comparative studies. Journal of the Royal Society Interface, 3(9), 507–514. 10.1098/rsif.2005.0110 PMC1664642 16849247

[jfb70593-bib-0067] Vincent, A. C. (1996). The international trade in seahorses.

[jfb70593-bib-0068] Vincent, A. C. , Foster, S. J. , & Koldewey, H. J. (2011). Conservation and management of seahorses and other Syngnathidae. Journal of Fish Biology, 78(6), 1681–1724. 10.1111/j.1095-8649.2011.03003.x 21651523

[jfb70593-bib-0069] Woods, C. M. C. (2000). Preliminary observations on breeding and rearing the seahorse Hippocampus abdominalis (Teleostei: Syngnathidae) in captivity. New Zealand Journal of Marine and Freshwater Research, 34(3), 475–485. 10.1080/00288330.2000.9516950

