# Supplementary material for: Early life history of the Korean seahorse, Hippocampus haema: Evidence for direct settlement and ontogenetic shifts in snout morphology
Source: J Fish Biol. 2026 Jul 24;109(4):2116–29. doi: 10.1111/jfb.70593 (PMC13675497; doi:10.1111/jfb.70593)
Supplement: Supplementary file 1 [file JFB-109-2116-s001.docx]

**Supplemental Materials**

**Early Life History of the Korean Seahorse, *Hippocampus haema*: Evidence for Direct Settlement and Ontogenetic Shifts in Snout Morphology**

**Authors:** Jaehwan Juan Lee^1, †^ and Jin-Koo Kim^1, *^

**Affiliations:**

^1^Department of Marine Biology, Pukyong National University, Busan 48513, Korea

^†^Present address: School of Biological Sciences, University of Canterbury, Christchurch 8140, New Zealand

Figure S1. Photograph of newborn *Hippocampus haema*. The newborn individual coiled its prehensile tail around an object and attached to it immediately after release.

Table S1. Individual morphometric measurements (mm) of Hippocampus haema across developmental stages. Note: NA indicates that snout depth could not be measured in specimens collected five days before release.

| **ID** | **Stage** | **Standard length (mm)** | **Head length (mm)** | **Trunk length (mm)** | **Tail length (mm)** | **Snout length (mm)** | **Snout depth (mm)** | **Eye diameter (mm)** |
| --- | --- | --- | --- | --- | --- | --- | --- | --- |
| PKUI829-1 | 5 days before release | 5.934 | 1.067 | 1.817 | 3.221 | 0.250 | NA | 0.422 |
| PKUI829-2 | 5 days before release | 5.964 | 1.404 | 1.683 | 2.888 | 0.280 | NA | 0.538 |
| PKUI829-3 | 5 days before release | 7.290 | 1.804 | 1.905 | 3.642 | 0.421 | NA | 0.698 |
| PKUI829-4 | 5 days before release | 8.942 | 1.667 | 2.231 | 4.956 | 0.396 | NA | 0.655 |
| PKUI829-5 | 5 days before release | 6.505 | 1.425 | 1.544 | 3.488 | 0.259 | NA | 0.580 |
| PKUI830-1 | Day of Birth | 11.697 | 2.498 | 2.741 | 6.464 | 0.700 | 0.555 | 0.772 |
| PKUI830-2 | Day of Birth | 12.394 | 2.352 | 2.856 | 6.304 | 0.685 | 0.612 | 0.821 |
| PKUI830-3 | Day of Birth | 11.533 | 2.392 | 2.569 | 6.495 | 0.677 | 0.571 | 0.753 |
| PKUI830-4 | Day of Birth | 11.975 | 2.646 | 2.532 | 6.772 | 0.785 | 0.588 | 0.800 |
| PKUI830-5 | Day of Birth | 11.821 | 2.629 | 2.500 | 6.405 | 0.788 | 0.574 | 0.812 |
| PKUI831-1 | 4 days after release | 14.152 | 3.202 | 4.075 | 6.875 | 0.897 | 0.612 | 0.944 |
| PKUI831-2 | 4 days after release | 14.665 | 3.376 | 4.020 | 7.269 | 0.993 | 0.644 | 0.976 |
| PKUI831-3 | 4 days after release | 15.355 | 3.569 | 4.128 | 7.658 | 0.975 | 0.650 | 1.055 |
| PKUI831-4 | 4 days after release | 15.307 | 3.379 | 4.070 | 7.858 | 1.004 | 0.640 | 1.021 |
| PKUI831-5 | 4 days after release | 15.238 | 3.362 | 4.406 | 7.470 | 0.950 | 0.632 | 1.022 |
| PKUI832-1 | 6 days after release | 15.249 | 3.093 | 3.612 | 8.429 | 0.862 | 0.642 | 0.998 |
| PKUI832-2 | 6 days after release | 15.507 | 3.283 | 3.681 | 8.597 | 1.170 | 0.631 | 0.931 |
| PKUI832-3 | 6 days after release | 17.044 | 3.501 | 4.227 | 9.481 | 1.098 | 0.663 | 1.025 |
| PKUI832-4 | 6 days after release | 14.711 | 2.973 | 3.601 | 8.076 | 0.752 | 0.644 | 0.899 |
| PKUI832-5 | 6 days after release | 15.315 | 3.229 | 3.712 | 8.359 | 0.997 | 0.609 | 0.941 |
| PKUI833-1 | 8 days after release | 17.429 | 3.530 | 4.352 | 9.639 | 1.235 | 0.638 | 0.988 |
| PKUI833-2 | 8 days after release | 17.329 | 3.561 | 4.360 | 9.384 | 1.229 | 0.650 | 0.981 |
| PKUI833-3 | 8 days after release | 17.585 | 3.496 | 4.521 | 9.645 | 1.230 | 0.669 | 0.953 |
| PKUI833-4 | 8 days after release | 18.627 | 3.767 | 4.468 | 10.374 | 1.284 | 0.697 | 1.033 |
| PKUI833-5 | 8 days after release | 16.989 | 3.685 | 4.224 | 9.163 | 1.239 | 0.675 | 1.005 |
| PKUI834-1 | 15 days after release | 17.979 | 3.671 | 4.567 | 9.811 | 1.243 | 0.646 | 1.023 |
| PKUI834-2 | 15 days after release | 17.739 | 3.395 | 4.299 | 10.132 | 1.203 | 0.651 | 0.954 |
| PKUI834-3 | 15 days after release | 17.360 | 3.553 | 4.162 | 9.642 | 1.247 | 0.643 | 0.947 |
| PKUI834-4 | 15 days after release | 17.730 | 3.580 | 4.547 | 9.698 | 1.271 | 0.640 | 0.955 |
| PKUI834-5 | 15 days after release | 18.966 | 3.919 | 4.696 | 10.467 | 1.298 | 0.699 | 1.100 |
| PKUI835-1 | 20 days after release | 21.711 | 4.230 | 5.470 | 12.041 | 1.479 | 0.701 | 1.112 |
| PKUI835-2 | 20 days after release | 21.171 | 3.904 | 5.675 | 11.631 | 1.410 | 0.701 | 1.122 |
| PKUI835-3 | 20 days after release | 25.870 | 4.719 | 6.833 | 14.591 | 1.709 | 0.850 | 1.353 |
| PKUI835-4 | 20 days after release | 21.852 | 4.309 | 5.156 | 12.367 | 1.477 | 0.721 | 1.150 |
| PKUI835-5 | 20 days after release | 21.870 | 4.324 | 5.623 | 12.052 | 1.496 | 0.741 | 1.157 |
| PKUI836-1 | 40 days after release | 22.778 | 4.667 | 5.702 | 12.408 | 1.714 | 0.756 | 1.250 |
| PKUI836-2 | 40 days after release | 23.694 | 5.054 | 5.917 | 12.723 | 1.743 | 0.820 | 1.340 |
| PKUI836-3 | 40 days after release | 25.576 | 5.586 | 6.317 | 13.673 | 1.859 | 0.911 | 1.370 |
| PKUI836-4 | 40 days after release | 27.219 | 5.702 | 6.896 | 14.621 | 1.965 | 0.931 | 1.398 |
| PKUI836-5 | 40 days after release | 28.933 | 5.818 | 7.527 | 15.588 | 2.130 | 0.952 | 1.520 |

Table S2. Model comparison and breakpoint analysis for morphometric traits of *Hippocampus haema*. Linear, power function, and segmented regression models were compared using AICc and R² values. Breakpoints were tested using Davies’ test. A dash indicates that no statistically significant breakpoint was detected. The asterisk indicates statistical significance at p < 0.05.

| **Trait** | **n** | **Linear AICc** | **Power AICc** | **Segmented AICc** | **Linear R2** | **Power R2** | **Segmented R2** | **Davies p** | **Breakpoint**  **(mm SL)** |
| --- | --- | --- | --- | --- | --- | --- | --- | --- | --- |
| Head length | 40 | -8.133 | -8.547 | -4.690 | 0.967 | 0.967 | 0.968 | 0.532 | - |
| Trunk length | 40 | 6.601 | 6.021 | 7.256 | 0.972 | 0.973 | 0.975 | 0.176 | - |
| Tail length | 40 | 28.897 | 29.267 | 31.995 | 0.990 | 0.989 | 0.990 | 0.588 | - |
| Snout length | 40 | -91.776 | -86.932 | -87.319 | 0.976 | 0.973 | 0.976 | 1 | - |
| Snout depth | 35 | -139.040 | -130.307 | -167.827 | 0.904 | 0.877 | 0.964 | <0.001* | 21.274 |
| Eye diameter | 40 | -113.353 | -111.650 | -113.989 | 0.945 | 0.943 | 0.953 | 0.055 | - |
